# Impact on disease-free survival of the duration of ovarian function suppression, as postoperative adjuvant therapy, in premenopausal women with hormone receptor-positive breast cancer: a retrospective single-institution study

**DOI:** 10.1007/s12282-018-0836-x

**Published:** 2018-01-22

**Authors:** Yukinori Ozaki, Yuko Tanabe, Nobuko Tamura, Takuya Ogura, Chihiro Kondoh, Yuji Miura, Kenichi Yoshimura, Hidetaka Kawabata, Toshimi Takano

**Affiliations:** 1 Department of Medical Oncology, Toranomon Hospital, 2-2-2 Toranomon, Minato-ku, Tokyo, 105-8470 Japan; 2 Department of Breast and Endocrine Surgery, Toranomon Hospital, 2-2-2 Toranomon, Minato-ku, Tokyo, 105-8470 Japan; 3 Innovative Clinical Research Center, Kanazawa University Hospital, Kakuma-city, Kanazawa, Ishikawa 920-8641 Japan

**Keywords:** Hormone receptor-positive, Breast cancer, Premenopausal, Ovarian function suppression, Duration, Propensity

## Abstract

**Introduction:**

Although tamoxifen (TAM) plus ovarian function suppression (OFS) is considered as a standard adjuvant treatment for premenopausal women with hormone receptor-positive breast cancer, the optimal duration of OFS has not yet been established. This retrospective study was designed to assess the duration of OFS and the impact of the duration of OFS on the DFS in these patients.

**Methods:**

We retrospectively reviewed the data of premenopausal patients with breast cancer who received TAM + OFS (goserelin or leuprorelin) as adjuvant therapy between February 2004 and June 2015. The primary analysis was a comparison of the disease-free survival (DFS) between patients who received OFS for 3 years or less (OFS ≤ 3 years group) and those who received OFS for longer than 3 years (OFS > 3 years group).

**Results:**

We analyzed the data of 215 premenopausal patients diagnosed as having hormone receptor-positive breast cancer. A propensity score-matched model showed the absence of any significant difference in the DFS between the OFS ≤ 3 years group and OFS > 3 years group (6-year DFS rate, 93.2 vs. 94.0%; log-rank test *p* = 0.767).

**Conclusions:**

Our data showed that among premenopausal women with hormone receptor-positive breast cancer who received TAM + OFS as adjuvant endocrine therapy, there was no significant difference in the DFS between the OFS ≤ 3-year group and OFS > 3-year group. A randomized trial is needed to establish the optimal duration of OFS for these patients.

## Introduction

Tamoxifen (TAM) plus ovarian function suppression (OFS) is one of the standard adjuvant treatments for premenopausal women with hormone receptor-positive breast cancer. Although some prospective studies have suggested that adjuvant TAM plus OFS provides a disease-free survival (DFS) benefit as compared to TAM alone, there is insufficient evidence as to the optimal duration of OFS needed in patients receiving adjuvant OFS in combination with TAM [1–3].

The prognosis of premenopausal women with hormone receptor-positive breast cancer is worse than that of postmenopausal women with breast cancer [4, 5]. The Suppression of Ovarian Function Trial (SOFT), a randomized phase III trial conducted by The International Breast Cancer Study Group (IBCSG), showed significant risk reduction, in terms of the DFS, obtained with the addition of OFS to TAM in women who were at a sufficient risk for recurrence to warrant adjuvant chemotherapy and who remained premenopausal until the end of the adjuvant chemotherapy; however, overall analysis of the patients in this trial failed to reveal significant benefit of the addition of OFS to TAM [6]. Therefore, the clinical benefit/additional toxicity balance of the addition of OFS to TAM should be carefully weighed.

The International Breast Cancer Study Group Trial VIIIA showed a significant benefit of the addition of OFS for 2 years to adjuvant chemotherapy in premenopausal women with lymph node-negative hormone receptor-positive breast cancer [7]. In the Austrian Breast and Colorectal Cancer Study Group Trial, while use of OFS for 3 years after adjuvant chemotherapy failed to show significant benefit in the overall patient population, a significant reduction in the risk of recurrence was reported in patients < 40 years of age [8]. Several meta-analyses have suggested that addition of OFS to TAM improved the DFS as compared to TAM alone in premenopausal patients younger than 40 years old; the duration of OFS in these studies varied from 2 to 3 years [9]. Based on the results of the SOFT trial, the American Society of Clinical Oncology clinical practice guideline recommends OFS for duration of 5 years [10]. In one randomized controlled pilot study conducted to determine the optimal duration of OFS, the survival benefit obtained with adjuvant leuprorelin treatment was similar between premenopausal patients treated for longer than 3 years and those treated for 2 years [11]. However, there are few data on the impact of the duration of OFS as adjuvant endocrine therapy, and the optimal duration of OFS has not yet been clearly established.

We conducted a single-institutional retrospective study to compare the DFS between patients who had received OFS for 3 years or less and those who had received OFS for longer than 3 years as adjuvant therapy. A propensity score-matched model was used for the analysis to minimize the effects of confounding factors.

## Patients and methods

### Patients

This study was designed as a retrospective review of the medical records of premenopausal patients with breast cancer who had received TAM plus OFS (goserelin or leuprorelin) as adjuvant therapy between February 2004 and June 2015 at Toranomon Hospital. The key inclusion criteria were as follows: (1) premenopausal women who had been diagnosed as having invasive breast carcinoma, Stage I–III, according to the American Joint Committee on Cancer (AJCC) staging system, Seventh Edition [12]; (2) patients with regular menstrual cycles or premenopausal hormone levels after chemotherapy; (3) patients who had undergone partial mastectomy or mastectomy and sentinel lymph node biopsy and/or axillary lymph node dissection; (4) patients in whom tumor immunohistochemistry (IHC) showed positive results for ER and/or PgR; (5) patients who had received standard adjuvant endocrine therapy with tamoxifen plus goserelin or leuprorelin. This study did not include patients who received bilateral salpingo-oophorectomy, because it was rarely performed as OFS in our institution.

Approval for this retrospective study was obtained from the Ethics Committee of Toranomon Hospital.

Clinicopathologic data were collected from the medical records, including the age, ER, PgR, HER2 and Ki67 status, nuclear grade, clinical and pathological stage, details of the neo-adjuvant/adjuvant chemotherapy given, and duration of OFS and DFS. Patients were defined as ER- and/or PgR- positive when the tumor IHC showed an Allred score for ER and/or PgR of ≥ 2. The tumor HER2 status was defined as positive when IHC for HER2 showed a 3 + or 2 + score, with confirmation by fluorescence in situ hybridization.

### Treatment

All patients received adjuvant hormone therapy with oral TAM at the dose of 20 mg daily for longer than 5 years. OFS was accomplished with a subcutaneous depot injection of goserelin acetate (Zoladex, AstraZeneca) at the dose of 3.6 mg once every month or 10.8 mg once every 3 months, or leuprorelin acetate (Leuplin, Takeda) at the dose of 3.75 mg once every month or 11.25 mg once every 3 months. The duration of TAM treatment and OFS was left to the discretion of the treating physician. The duration of OFS was calculated from the date of initiation to the date of completion of treatment with a luteinizing hormone-releasing hormone analogue or to a DFS event.

### Endpoints and statistical analysis

The primary endpoint of the study was the DFS, defined as the length of survival time after primary treatment without any signs or symptoms of cancer, including loco-regional recurrence, contralateral breast cancer, distant recurrence, and second malignancy. Loco-regional recurrence was defined as pathologically confirmed disease recurrence in the ipsilateral chest wall, or within the supra-clavicular, subclavian, ipsilateral axillary, or ipsilateral internal mammary lymph nodes. The secondary endpoint was the overall survival (OS). The primary analysis was a comparison of the DFS between patients who had received OFS for 3 years or less (OFS ≤ 3-year group) and those who had received OFS for longer than 3 years (OFS > 3-year group), using the Cox proportional hazards model, propensity score-matched model. Because of the non-randomized design of this retrospective study, we used propensity score-matched models to reduce the influence of differences in the confounding factors between the OFS ≤ 3 years and OFS > 3-year groups. Propensity score was calculated based on the age, pT, pN, HER2 status, and perioperative chemotherapy. These data were analyzed using the JMP ver.12.0.1 (2015 SAS Institute Inc.). The Kaplan–Meier method was used for the survival analyses, and the log-rank test was used to compare the survivals between two groups, and univariate and multivariate analyses were conducted using the Cox proportional hazards model. Differences between the two groups were assessed by the Chi-square test. *p* values less than 0.05 were considered to denote significance.

## Results

The medical records of patients were searched from the database of our institution using the search terms “breast cancer” and “goserelin acetate” or “leuprorelin acetate” for the period between February 2004 and June 2015, and 532 patients were detected. Among these, 215 patients met the inclusion criteria for this study. The median follow-up duration of the patients was 71 months (range 20.3–144.3). The patient characteristics are shown in Table 1. The median age was 42 years (range 24–53). The number of patients with pathological (p) T0–T1 was 149 (69%), and that of patients with pN0 was 164 (76%). The HER2 status was positive in 15 (7%) patients. Neo-adjuvant or adjuvant chemotherapy had been administered in 66 (31%) patients. The regimens used for the neo-adjuvant or adjuvant chemotherapy were fluorouracil + epirubicin + cyclophosphamide (FEC), conventional doxorubicin + cyclophosphamide (AC) or dose-dense AC, followed by weekly paclitaxel or triweekly docetaxel. Trastuzumab was given for patients with an HER2-positive disease status. Of all the patients, 152 (71%) received OFS for ≤ 3 years and 63 (29%) received OFS > 3 years.

**Table 1:** Patient characteristics

Characteristics	No.	%
Age	*N* = 215	
Median 42 years (range 24–53)		
≤ 40	86	40
> 40	129	60
pT stage		
T0	1	0.5
Tis	2	1
T1	146	68
T2	52	24
T3	12	6
T4	2	1
pN stage		
N0	164	76
N1	42	20
N2	8	4
N3	1	0.5
Ki-67		
Median 8 (range 0–80) (%)		
0–20	112	52
> 20	20	9
Unknown	83	39
HER 2 status		
Positive	15	7
Negative	200	93
Nuclear grade		
NG1	136	63
NG2	49	23
NG3	17	8
Unknown	11	5
Neo-adjuvant/adjuvant chemotherapy		
Yes	66	31
No	149	69
Duration of OFS		
Median 896 (range 104–3676) (day)		
≤ 3 years	152	71
> 3 years	63	29

**Fig. 1:**
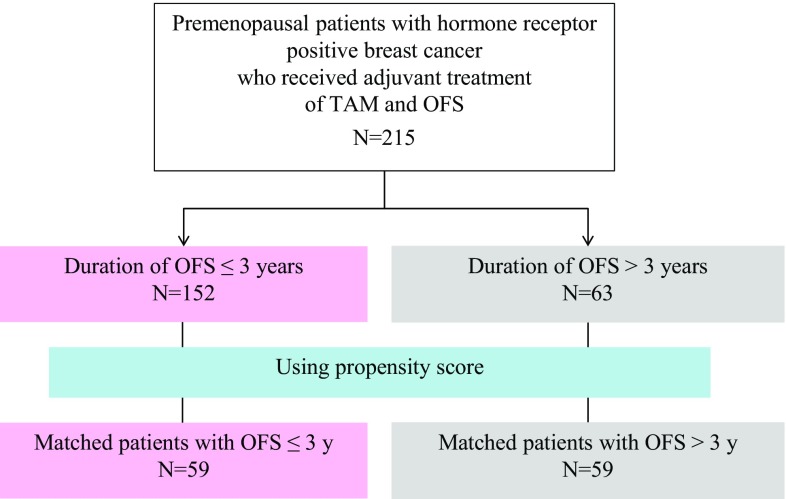
Patient diagram. Based on the propensity score, the OFS ≤ 3 years and OFS > 3-year groups had 59 matched patients each that were included for the primary analysis

Propensity score matching was used to make the two groups with similar in terms of the baseline characteristics, based on the age, pT stage, pN stage, HER2 status, and the neo-adjuvant or adjuvant chemotherapy regimens used. The patient diagram is shown in Fig. 1. Based on the propensity score, the OFS ≤ 3 years and OFS > 3-year groups had 59 matched patients each that were included for the primary analysis. The patient characteristics in the two groups are shown in Table 2. The age (≤ 40/> 40 years) (*p* = 0.852), pT (≤ 2 cm/> 2 cm) (*p* = 0.853), pN status (negative/positive) (*p* = 0.849), HER2 status (negative/positive) (*p* = 0.507), nuclear grade (*p* = 0.922), and chemotherapy regimens employed (*p* = 0.577) were well balanced between the OFS ≤ 3 years and OFS ≤ 3-year groups.

**Table 2:** Patient characteristics in the propensity score-matched groups

Characteristics	OFS ≤ 3 years		OFS > 3 years		*p*
	*N* = 59	(%)	*N* = 59	(%)	
Age					
≤ 40 years	25	42	26	44	
> 40 years	34	58	33	56	0.852
pT					
≤ 2 cm	31	53	32	54	
> 2 cm	28	47	27	46	0.853
pN					
Negative	36	61	37	63	
Positive	23	39	22	37	0.849
HER 2 status					
Positive	4	7	6	10	
Negative	55	93	53	90	0.507
Nuclear grade					
NG1	33	56	36	61	
NG2,3	20	34	21	36	0.922
Neo-adjuvant/adjuvant chemotherapy					
Yes	35	59	32	54	
No	24	41	27	46	0.577

**Fig. 2:**
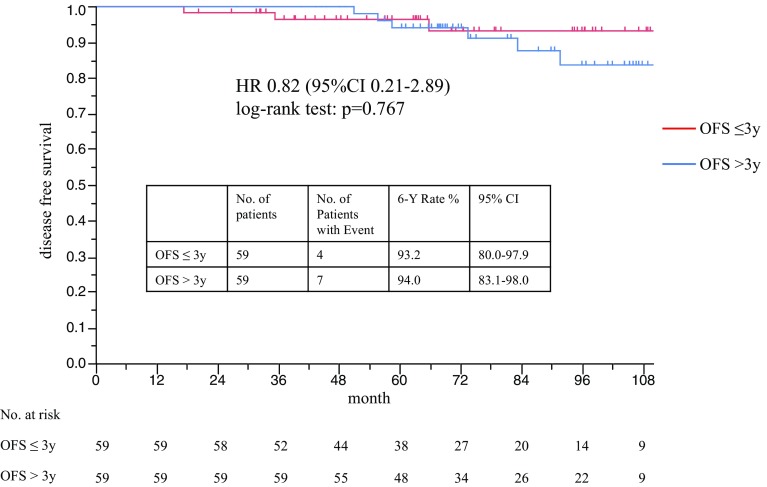
Disease-free survival in propensity matched patients. Kaplan–Meier curve of DFS between the OFS ≤ 3 years and OFS > 3-year groups (hazard ratio 0.82, 95% CI 0.21–2.89, log-rank test *p* = 0.767). The DFS rate at 6 years was 93.2% in the OFS ≤ 3-year group and 94.0% in the OFS > 3-year group

The primary analysis showed the absence of any significant difference in the DFS between the OFS ≤ 3 years and OFS > 3-year groups (hazard ratio 0.82, 95% CI 0.21–2.89, log-rank test *p* = 0.767). The DFS rate at 6 years was 93.2% in the OFS ≤ 3 years group and 94.0% in the OFS > 3 years group (Fig. 2). The results of univariate Cox regression analysis conducted to identify factors influencing the DFS in the matched patients are shown in Table 3. The risk ratio (RR) of age ≤ 40 and pT > 2 cm was significantly greater, at 4.25 (95% CI 1.15–20.30, *p* = 0.029) and > 20 (*p* < 0.0001), respectively. Node-positive status (RR 1.21, 95% CI 0.33–4.37), the chemotherapy regimen used (RR 2.19, 95% CI 0.60–10.30), nuclear grade > 1 (RR 1.13, 95% CI 0.27–4.29), and HER2-positive status (RR 1.44, 95% CI 0.07–7.87) were not identified as having any significant influence on the prognosis in either of the groups. Multivariate Cox regression analysis conducted to identify factors influencing the DFS in the matched patients showed that OFS ≤ 3 years was not a prognostic factor (RR 1.16, 95% CI 0.29–4.18), while age ≤ 40 years (RR 4.01, 95% CI 1.08–19.31, *p* = 0.038) and pT > 2 cm (RR > 20, *p* < 0.0001) were significant prognostic factors (Table 4).

**Table 3:** Univariate cox regression analysis to identify factors influencing the DFS in propensity score-matched patients

	Risk ratio	*p*	95% CI
OFS ≤ 3 years	0.82	0.767	0.21–2.89
Age ≤ 40 years	4.25	0.029	1.15–20.30
pT > 2 cm	> 20	< 0.0001	NE
N-positive	1.21	0.767	0.33–4.37
Chemotherapy	2.19	0.238	0.60–10.30
Nuclear grade > 1	1.13	0.858	0.27–4.29
HER2-positive	1.44	0.741	0.07–7.87

**Table 4:** Multivariate cox regression to identify factors influencing the DFS in the matched patient groups

	Risk ratio	*p*	95% CI
OFS ≤ 3 years	1.16	0.822	0.29–4.18
Age ≤ 40 years	4.01	0.038	1.08–19.31
pT > 2 cm	> 20	< 0.0001	NE

For all the included patients, the Cox proportional hazards model using the duration of OFS and propensity score showed that the RR of OFS ≤ 3 years was 0.82 (*p* = 0.750), which was not significantly different from the RR in the OFS > 3-years group.

**Fig. 3:**
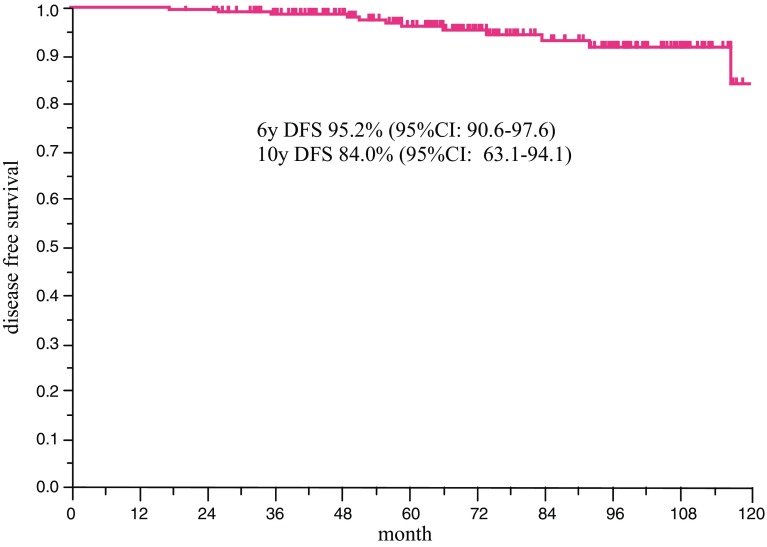
Disease-free survival in all patients. Kaplan–Meier curve of DFS in all patients (*n* = 215). The DFS rate at 6 years was 95.2 and 84.0% at 10 years

The 6-year DFS of the patients, overall, was 95.2% (95% CI 90.6–97.6), and the 10-year DFS was 84.0% (95% CI 63.1–94.2) (Fig. 3). In patients who were younger than 40 years of age and/or had received perioperative chemotherapy, the 6-year DFS was 93.4% (95% CI 86.1–97.0). The RR of OFS ≤ 3 years in these patients was 0.43 (*p* = 0.217), which was not significant.

A total of 13 DFS events were detected in the study population (6 events in the OFS ≤ 3-year group, and 7 events in the OFS > 3 years group), and 2 patients died of the breast cancer. OS as a secondary endpoint was not analyzed because of the short follow-up period and infrequent OS events.

## Discussion

Analyses of the retrospective data using propensity score matching showed no statistically significant difference in the DFS between patients who had received adjuvant endocrine therapy plus OFS for ≤ 3 years and those who had received it for > 3 years. Although prospective studies have suggested a role of OFS in premenopausal women with hormone receptor-positive breast cancer, few studies have compared the clinical benefit of OFS for ≤ 3 and > 3 years. To the best of our knowledge, this is the first retrospective study to compare the impact on the DFS of OFS for ≤ 3 years and OFS for > 3 years administered in combination with TAM as adjuvant therapy in premenopausal breast cancer patients.

The duration of OFS had been left to the discretion of the attending physicians and the patients’ wishes, depending on the biological and clinical features of the disease. Before publication of the SOFT trial, some patients received OFS for less than 3 years, although patients who were thought by their attending physicians as being at a high risk for recurrence received OFS for longer than 3 years at our institution. Propensity score matching was used to minimize confounding factor bias in this retrospective study. The DFS was not significantly different among the propensity score-matched model and Cox proportional hazards model, suggesting that OFS ≤ 3 years was as effective as OFS > 3 years in this population.

Adverse events, including hot flushes, depression, musculoskeletal symptoms, and quality of life (QOL) data, were not evaluated in this study. The health-related QOL data of the E-3193 trial showed that addition of OFS to TAM as adjuvant therapy resulted in a more significant increase of the menopausal symptoms and sexual dysfunction as compared to the symptoms observed in the women receiving TAM alone [13]. In the SOFT trial, hot flushes, sweating, decreased libido, vaginal dryness, insomnia, depression, musculoskeletal symptoms, hypertension, and glucose intolerance were reported more frequently in the 5-year OFS plus TAM group than in the TAM alone group, and the frequency of ≥ grade 3 adverse events was 31.3% in the OFS plus TAM group and 23.7% in the TAM alone group [6]. The SOFT trial also showed that the rate of non-adherence to OFS increased gradually with time, being 9.2, 14.9, 18.3, and 21.9% at 1, 2, 3, and 4 years, respectively. The estimated cost of OFS with goserelin or leuprorelin is about $2320 per person-year. The balance among the toxicity, cost, and clinical benefit should be carefully taken into consideration in relation to OFS as adjuvant therapy in premenopausal breast cancer patients.

### Study limitations

This retrospective study was conducted without randomization in a small patient population at a single site. The number of events was relatively small. Most of the patients had low-risk disease with node-negative or no chemotherapy, which suggests the difficulty in evaluating the benefit of OFS. Premenopausal estradiol levels or menstruation after neo-adjuvant/adjuvant chemotherapy were not confirmed in the patients. Use of an aromatase inhibitor with OFS was not evaluated, because aromatase inhibitor agents are not yet approved for administration to premenopausal women in Japan.

Although adjuvant hormone therapy with 5-year OFS plus TAM is recommended for premenopausal patients with hormone receptor-positive breast cancer who are at a sufficient risk for recurrence to warrant adjuvant chemotherapy and who are expected to remain premenopausal until the end of the adjuvant chemotherapy, the optimal duration of OFS should be further investigated with a detailed assessment of the adverse events, QOL, financial burden with careful consideration given to the lack of definite evidence of a meaningful clinical benefit, i.e., impact, of this treatment on the disease outcome. Thus, our results should be confirmed in a prospective, randomized controlled trial including a larger patient population.

## Conclusions

This study suggests that in premenopausal women with hormone receptor-positive breast cancer, the DFS of the patients did not differ significantly between patients receiving OFS for ≤ 3 years and those receiving OFS for > 3 years. A randomized trial is needed to establish the optimal duration of OFS for these patients.

## Abbreviations

TAM: Tamoxifen
OFS: Ovarian function suppression
DFS: Disease-free survival
AJCC: American Joint Committee on Cancer
IHC: Immunohistochemistry
OS: Overall survival
FEC: Fluorouracil + epirubicin + cyclophosphamide
AC: Doxorubicin + cyclophosphamide
RR: Risk ratio
QOL: Quality of life
